# Triptans attenuate capsaicin-induced CREB phosphorylation within the trigeminal nucleus caudalis: a mechanism to prevent central sensitization?

**DOI:** 10.1007/s10194-011-0352-2

**Published:** 2011-05-29

**Authors:** Dimos D. Mitsikostas, Yolande E. Knight, Michele Lasalandra, Nikolaos Kavantzas, Peter J. Goadsby

**Affiliations:** 1 Headache Group, Department of Neurology, University of California, San Francisco, San Francisco, CA USA; 2 Neurology Department, Athens Naval Hospital, 77A Vas. Sofias Avenue, 11521 Athens, Greece; 3 1st Department of Pathology, Medical School, Athens National University, Athens, Greece

**Keywords:** CREB, Sumatriptan, Naratriptan, Capsaicin, Migraine, Sensitization

## Abstract

The c-AMP-responsive element binding protein (CREB) and its phosphorylated product (P-CREB) are nuclear proteins expressed after stimulation of pain-producing areas of the spinal cord. There is evidence indicating that central sensitization within dorsal horn neurons is dependent on P-CREB transcriptional regulation. The objectives of the study were to investigate the expression of P-CREB in cells in rat trigeminal nucleus caudalis after noxious stimulation and to determine whether pre-treatment with specific anti-migraine agents modulate this expression. CREB and P-CREB labelling was investigated within the trigeminal caudalis by immunohistochemistry after capsaicin stimulation. Subsequently, the effect of i.v. pre-treatment with either sumatriptan (*n* = 5), or naratriptan (*n* = 7) on P-CREB expression was studied. Five animals pre-treated with i.v. normal saline were served as controls. CREB and P-CREB labelling was robust in all animal groups within Sp5C. Both naratriptan and sumatriptan decreased P-CREB expression (*p* = 0.0003 and 0.0013) within the Sp5C. Triptans attenuate activation of CREB within the central parts of the trigeminal system, thereby leading to potential inhibition of central sensitization. P-CREB may serve as a new marker for post-synaptic neuronal activation within Sp5C in animal models relevant to migraine.

## Introduction

The c-AMP-responsive element binding protein (CREB) is a transcription factor that regulates the expression of genes important for adaptive neuronal responses [1] as well as complex functions such as learning and memory [2]. Upon extracellular stimuli multiple kinases, such as protein kinase A, protein kinase C and casein kinase II, phosphorylate CREB at serine 133 (P-CREB) leading to activation of immediate early gene *c-fos* [3, 4]. Fos protein expression can then be used as a marker of neuronal activation within brainstem and spinal nociceptive pathways [5]. CREB also target other genes, such as brain-derived neurotrophic factor (BDNF), that is significantly involved in depression, serving as potential marker for treatment together with P-CREB [6].

Like *c-fos*, CREB signalling is also involved in pain processing, e.g. at the level of spinal cord in a study of tissue injury-induced inflammation and hyperalgesia [7]. Likewise, at the brainstem level, in vitro evidence suggests that P-CREB within the rat trigeminal ganglion is regulated by calcitonin gene-related peptide (CGRP) induced by activation of either the adenosine A_1_ receptor or the P2X3 receptor [8, 9]. In addition, it has been proposed that inhibition of CREB prior to its nuclear translocation may prevent the slowly developing onset of sensitization within the brainstem [9], as other studies indicate that central sensitization within the dorsal root neurons is mediated via P-CREB-mediated transcriptional regulation [10].

Allodynia has been recognized in migraine since the nineteenth century [11], with clinic- [12] and population- [13] based studies showing that it is seen in about two-thirds of patients. Allodynia is a clinical reflection of sensitization, and both central and peripheral sensitization are important insofar as they both influence attacks and perhaps disease progression [14, 15]. Sensitization of peripheral trigeminovascular neurons that innervate the meninges may be crucial for the development of throbbing in the initial phase of migraine following by sensitization of central trigeminal neurons within the trigeminal nucleus caudalis [16]. In previous work, sumatriptan did not change P-CREB induced by forskolin in cultured neurons taken from adult rat trigeminal ganglions, nor inhibit CGRP release at the same model, as may expected [8], although the role of CGRP on sensitization is unclear [17]. An interesting question remains as to whether triptans modulate phosphorylation of CREB within neurons of the trigeminal nucleus caudalis in vivo. We, therefore, mapped P-CREB expression within rat trigeminal nucleus caudalis after capsaicin stimulation of the trigeminovascular system, and determined the effect of triptans, serotonin 5-HT_1B/1D_ receptor agonists. We show capsaicin stimulation of the trigeminal system activates P-CREB within trigeminal nucleus caudalis and both sumatriptan and naratriptan inhibit induced P-CREB at doses relevant to clinical practice.

## Methods

Male Sprague–Dawley rats (240–300 g) were housed under diurnal lighting and allowed food and water ad libitum. All experiments were conducted consistent with the UK Home Office Animals (Scientific Procedures) Act (1986).

### CREB and P-CREB mapping with immunohistochemistry

Eight rats were anaesthetized with pentobarbital i.p. (60 mg/kg) and the femoral vein was cannulated for physiology and drug treatments. Ten minutes later two rats were killed (intact animals), whereas a craniotomy was performed in the remaining six animals. After craniotomy 0.1, 1 or 10 μM capsaicin in a cotton ball was directly applied onto the right middle meningeal artery (MMA) for 5 min (*n* = 2 for each capsaicin dose). Ten minutes after the end of stimulation period, the animals were killed by pentobarbital (100 mg/kg, i.v.) and perfused with PBS followed by paraformaldehyde. Brainstems were removed and kept in 30% sucrose until sectioning in a freezing microtome (5 μm) followed by immunohistochemistry (avidin–biotin procedure).

Several dilutions of P-CREB and CREB antibodies (Cell Signalling Technology, cat# 9191 and 9192, respectively) were tested to establish 1:100 as optimal. Biotinylated sheep anti-rabbit serum (vector) served as a secondary antibody (1:600). Tissues from two non-stimulated animals were used as controls. The brainstems from two other intact animals, where only anaesthesia followed by killing was performed after 30 min, were processed for immunohistochemistry to study the effect of surgery on CREB and P-CREB expression at a comparable timing. Two additional animals were treated as above following exactly the same procedure but killed 2 h after capsaicin stimulation (1 μM). Their brainstems were prepared for fos protein immunohistochemistry at the level of the trigeminal nucleus caudalis [18] to determine if the stimulation was sufficient to activate the trigeminal neurons. Although we refer to the labelling as CREB and P-CREB we cannot exclude cross-reactivity of the antibody so that CREB-like and P-CREB-like may be better terms.

### Pharmacological studies

The effect of i.v. pre-treatment with either sumatriptan 1 mg kg^−1^ (*n* = 5), or naratriptan 1 mg kg^−1^ (*n* = 7) on P-CREB expression within Sp5C was studied following the above described procedure. Five animals pre-treated with i.v. normal saline given at the same volume as the drugs served as controls.

### Physiology

Physiological monitoring was carried out in all animals, including arterial pressure (MABP), heart rate (HR) and rectal temperature.

### CREB and P-CREB immunoreactive cell counting (image analysis method)

Images were acquired using a Zeiss Axiolab microscope (Carl Zeiss GmbH Jena, Germany) with a mechanical stage, fitted with a Sony-iris CCD videocamera (Sony Corp. Tokyo, Japan). The video camera was connected to a PC (Pentium IV-based) loaded with appropriate image analysis software (Sigma Scan Pro 5.0, Science, Germany). Slides were examined at a magnification ×40. More specifically, in each case the cellular area of the trigeminal nucleus caudalis was selected according to the atlas of Paxinos and Watson [19] and then the number of P-CREB immunostaining cells were calculated via a semiautomatic procedure using the above-mentioned image analysis software. Prototypes of positively stained neurons were carefully selected by an expert (DDM) and then labelled cells were counted within the pre-defined areas by the software automatically. Counting was performed by an investigator blind to drug treatment (NK). Twelve coronal sections of the trigeminal nucleus caudalis were selected for counting from each animal; four consecutive slides at the obex level, four at level −2 mm and other four at the level −6 mm (C2) [20]. The mean number was calculated and used for statistics for each animal.

## Results

Blood pressure and heart rate (not shown) did not differ between animals, except that both the animals treated with high capsaicin dose (10 μM) died.

### CREB and P-CREB mapping with immunohistochemistry

**Fig. 1:**
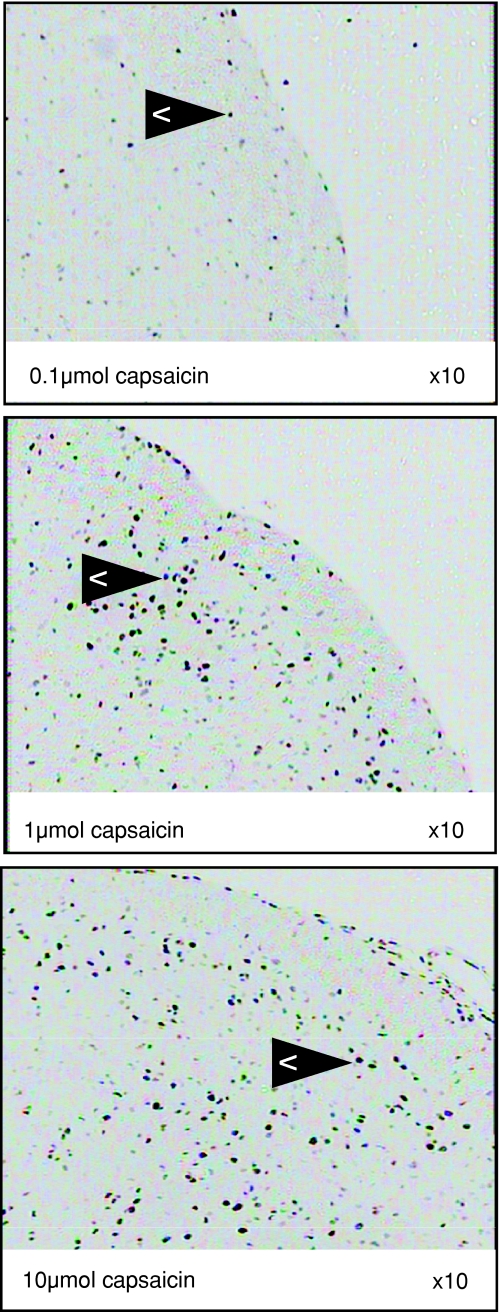
Micro-photographs show P-CREB expression within the rat trigeminal nucleus caudalis (Sp5C) after capsaicin stimulation (obex). Capsaicin applied onto the middle meningeal artery (MMA) for 5 min at three different doses (1, 0.1 and 10 μM, *n* = 2 for each dose). P-CREB-labelled neurons are shown (*arrows*) within the rat trigeminal nucleus caudalis (obex) 10 min after the end of stimulation. Avidin–biotin immunohistochemistry

In the intact animals CREB but not P-CREB induction was detected bilaterally within all parts of trigeminal nucleus caudalis (data not shown). After craniotomy, CREB was detected bilaterally within almost all the brainstem nuclei including the trigeminal nucleus caudalis, solitary tract, area postrema, lateral reticular nucleus, inferior olive nucleus, within all the laminae. P-CREB was not detected within the trigeminal nucleus caudalis. At the dose of 1 μM of capsaicin CREB labelling increased in all the above-mentioned nuclei and P-CREB labelling was found as well within the ipsilateral side of trigeminal nucleus caudalis laminae I and II (5–30 cells per section within the obex and the rostal parts down to C_2_ level). Neurons in lamina X were labelled as well, whereas only a few contralateral neurons were labelled at laminae I, II. Capsaicin 0.1 μM induced weak P-CREB labelling in cells within laminae I and II (1–5 neurons per section) (Fig. 1). Fos protein-labelled neurons were observed within Sp5C laminae I and II 2 h after 1 μmol capsaicin stimulation. Fos labelling was robust and clear. After capsaicin stimulation (0.1 and 1 μM) one animal died in each dose (mortality rate 10%). Therefore, the dose of 1 μM of capsaicin was selected for consecutive drug testing.

### Pharmacological studies

**Fig. 2:**
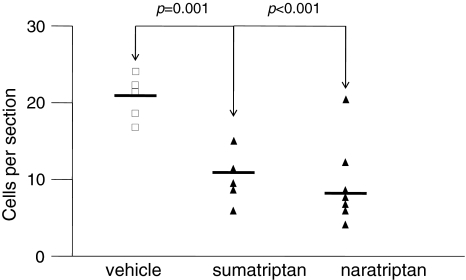
Treatment with both sumatriptan and naratriptan attenuates CREB activation within trigeminal nucleus caudalis. Sumatriptan (*n* = 5) and naratriptan (*n* = 7) at a dose of 1 mg kg^−1^ i.v. significantly decreased capsaicin (1 μmol) induced P-CREB expression within trigeminal nucleus caudalis compared to vehicle treated animals (*n* = 5)

**Fig. 3:**
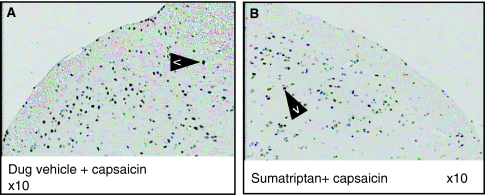
Micro-photographs show P-CREB expression within the rat trigeminal nucleus caudalis (Sp5C) after capsaicin stimulation and sumatriptan treatment (obex). Normal saline (*n* = 5) or sumatriptan 1 mg kg^−1^ (*n* = 5) was i.v. administered in rats and 10 min later 1 μM capsaicin applied onto the middle meningeal artery (stimulation lasted for 5 mins). Ten minutes after the end of stimulation the animals were killed and preceded for immunohistochemistry (avidin–biotin procedure). *Panel A* sample from a vehicle + capsaicin-treated animal. *Panel B* sample from a sumatriptan + capsaicin-treated (1 mg kg^−1^) animal. P-CREB labelled neurons are shown (*arrows*) within the rat trigeminal nucleus caudalis (obex)

Pre-treatment with i.v. sumatriptan and naratriptan at a dose of 1 mg kg^−1^ significantly decreased capsaicin-induced P-CREB expression within laminae I and II of trigeminal nucleus caudalis compared to vehicle pre-treated animals (Figs. 2, 3). Two treated animals died (mortality rate 10.5%). The physiological data were not different between three groups (data not shown).

## Discussion

We show here that stimulation of the peripheral parts of the rat trigeminovascular system, first-order trigeminal neurons, by capsaicin-induced CREB phosphorylation within the second-order neurons at the brainstem in the trigeminal nucleus caudalis. To our knowledge, this is the first in vivo study mapping the CREB and P-CREB expression within the trigeminal nucleus caudalis after noxious stimulus. Furthermore, the anti-migraine drugs sumatriptan and naratriptan prevented brainstem CREB activation.

### Methodological limitations

Preabsorption controls for anti-CREB and anti-P-CREB were not performed, to prove their specificity to the protein using the exact immunohistochemical protocol except for the addition of the immunizing peptide. However, we followed the manufacturer’s immunohistochemical protocols and the P-CREB labelling distribution within the pain-producing areas of the trigeminal system supports the specificity of the antibodies used. Several time points for CREB and P-CREB expression were not tested to see if the 10-min window was optimal for drug testing. We followed suggestions based on previous experiments [7, 21] showing that this time window, 10 min after stimulation of MMA to observe phosphorylation of CREB within trigeminal nucleus caudalis, was appropriate. The mortality rate was slightly high (10%) but acceptable. Finally, a TRPV1 antagonist could be used to show a reverse effect. We tested only one dose of triptans, not several to show a possible dose–response related effect. Also the effect of triptans to inhibit other stimuli (mechanical distension or thermal stimuli or other chemical stimuli) was not studied.

### CREB and P-CREB mapping within the trigeminal system

Capsaicin 1 μmol applied on the MMA-induced P-CREB immunohistochemistry within the ipsilateral brain stem trigeminal nucleus caudalis, as it has been shown with *c*-*fos* [5]. P-CREB within Sp5C was relatively pain-specific because of the particular distribution within the laminae I and II, that was clear for counting and with good signal to noise for drug testing. Physiological monitoring showed that the microsurgical procedure and the capsaicin stimulation were tolerated well by the pentobarbital anaesthetized rats. Previous in vitro experiments showed P-CREB expression within the rat trigeminal ganglion neurons induced by forskolin [8]. Recently, activation of CREB within mouse cultured trigeminal ganglion neurons was seen after treatment with CGRP via Ca^2+^-dependent mechanisms [9]. We had the opportunity to look for CREB and P-CREB expression within the ipsilateral trigeminal ganglia after capsaicin stimulation; we did not observe P-CREB. Apparently the CREB cascade exists in rat trigeminal ganglion cells, although how to induce it in vivo still remains unclear and needs further investigation.

### Triptan effects on P-CREB within trigeminal nucleus caudalis

Both sumatriptan and naratriptan prevented capsaicin-induced P-CREB within the trigeminal nucleus caudalis as previously shown with capsaicin-induced *c*-*fos* [22, 23] indicating that the P-CREB approach yields similar data. At the first neuron level, however, in vitro studies failed to show an effect of sumatriptan on forskolin-induced CREB activation [8], suggesting that sumatriptan triggers the suppressive transcriptional cascade only within the central parts of the trigeminal system, not in the periphery. No such model for *c*-*fos* exists to compare to, since *c*-*fos* is not expressed within trigeminal ganglion cells [5]. To better explore the effect of triptans on P-CREB, two drugs with somewhat different pharmacological properties have been tested: the hydrophilic sumatriptan, which does not penetrate the blood brain barrier in normal circumstances, and the tenfold more lipophilic naratriptan [24]. Because both drugs share almost similar affinity to 5-HT1 receptors, we used the same doses for both drugs. The clinically relevant doses are different although this may be simply a matter of absorption. Both triptans attenuated P-CREB regardless of their differences. It is important, however, to understand the molecular mechanism of the action of triptans on neurons. Triptans not only increase intracellular Ca^2+^ leading in inhibition of CGRP gene transcription [25, 26], but also down regulate gene transcription generally within trigeminal nucleus caudalis cells by blocking CREB phosphorylation and *c*-*fos* expression, resulting in depression of brainstem nociceptive neurons, as has been shown using electrophysiological methods [27, 28]. These findings may partially explain the effect of triptans on the central sensitization process seen in migraine.

### Central sensitization and P-CREB

It has been speculated that triptans can successfully abort migraine as long as central sensitization still depends on incoming signals from the periphery, but not after central sensitization becomes self-sufficient [29]. Since this hypothesis still lacks good documentation, it would be interesting to test the effect of triptans in vivo within the trigeminal ganglion neurons. However, only electrophysiological studies can be performed, and these techniques are limited to short lasting neuronal alterations. One of the aims of this study was to explore an in vivo approach to investigate the longer time course effects of triptans within both trigeminal ganglion and trigeminal nucleus caudalis neurons, such as effects on gene transcription. Unfortunately trigeminal ganglion cells did not show sufficient labelling to explore this question further. Yet, the findings of our study may serve as vehicle to better understand the effect of triptans on slowly developing central sensitization within trigeminal nucleus caudalis.

Gene transcription is crucial for long lasting neuronal changes triggered by several extracellular signals. In the case of CREB, multiple molecules or proteins, such as CGRP [9], lead to its phosphorylation, via activation of several kinases [1, 30–32]. After phosphorylation CREB activates other immediate early genes like *c*-*fos*, molecules important for synaptic function like brain-derived neurotrophic factor and neuronal nitric oxide [33], which are all involved in pain transmission. Thus, selective blocking of CREB activation before its nuclear translocation would be important in inhibiting pain signalling acutely and also in blocking the sensitization cascade triggered by P-CREB, therefore reducing escalation of pain signalling [9]. In other words this would be pivotal for reducing the escalation/persistence of pain due to central sensitization, such as in migraine. In addition, P-CREB may also serve as marker of neuronal presynaptic activation within the trigeminovascular system in animal models of migraine biology, like fos protein.

### *C*-*fos* and P-CREB as markers for trigeminal caudalis activation in animal models of migraine biology

*C*-*fos* encodes a nuclear protein that regulates the transcription of other target genes and of its one promoter. Its detection within the brainstem and spinal cord neurons is the best studied technique to map the postsynaptic nociceptive pathways in numerous cephalic pain models [5, 18, 34]. In the field of migraine research fos protein immunoreactivity offers a method to identify subpopulations of neurons activated in response to noxious stimuli and identify related nociceptive pathways [5]. The majority of studies have employed this technique to map neuronal activation not only throughout the trigeminovascular system [35–39], but also higher structures involved in the ascending and descending modulatory control of pain [6, 40–42], thus, greatly enhancing our understanding of the pathophysiology of migraine. As with other models of the components of migraine the use of fos expression has certain limitations [43]. The major one being the model can only be as good as the stimulus since it is the stimulus that drives the expression of fos protein. Several intracranial structures have been stimulated chemically or electrically to induce fos within trigeminal nucleus caudalis, including the meninges [18, 20, 22, 23], trigeminal ganglion [44], the superior sagittal sinus [35] and middle meningeal artery [45]. It seems that the activation of specific structures of the trigeminovascular system (e.g. the superior sagittal sinus) leads to more clinically relevant conclusions [35, 46]. It is noteworthy that induction of fos to quantifiable levels requires a strong consistent stimulation that is often not physiological [47, 48]. It must also be remembered that *c*-*fos* is not expressed in all neurons as with the dorsal root ganglion cells [34], thus, lack of fos protein expression does not equate to lack of neuronal activity. Technical difficulties in inducing *c*-*fos* expression within trigeminal nucleus caudalis are also important [49]. One final limitation of the model is seen via direct activation of the antinociceptive descending pathways, which elicits *c*-*fos* expression in spinal neurons even in the absence of any nociceptive input [50].

P-CREB on the other hand displays several advantages over *c*-*fos* as a neuronal marker of post-synaptic activation within trigeminal nucleus caudalis. So far, activation of CREB within the trigeminal nucleus caudalis, or trigeminal ganglion in vitro, has been seen only after specific activation of nociceptive neurons, thus P-CREB within laminae I and II seems to be a direct effect of the noxious stimulus used. In addition, the time window needed for phosphorylation of CREB is much shorter, up to 10 min, compared to 120 min needed for *c*-*fos* expression, leading potentially to lower animal mortality rates. Although only stimulation of middle meningeal artery has been used, theoretically all the other parts of the peripheral trigeminal system can be used to induce P-CREB. The fact that P-CREB expression has been detected within trigeminal ganglion, although only in vitro, raises new possibilities for pharmaocological studies within both the peripheral and central parts of the trigeminal system. Further investigation is required though to induce and map P-CREB in trigeminal ganglion cells in vivo.

## Conclusions

P-CREB is a transcriptional factor involved in sensitization of nociceptive cells. Noxious stimulation of peripheral parts of the trigeminal system induces P-CREB within trigeminal nucleus caudalis. Triptans inhibit this activation. Thus, triptans might prevent central sensitization by attenuating P-CREB-mediated transcriptional pathways. In addition, P-CREB may serve as a new marker for post-synaptic neuronal activation within trigeminal nucleus caudalis in animal models of migraine biology.
